# Factors associated with common mental disorders among farmers in a medium-sized municipality in Northeastern Brazil

**DOI:** 10.11606/s1518-8787.2022056003522

**Published:** 2022-08-02

**Authors:** Roberta Machado Alves, Emelynne Gabrielly de Oliveira Santos, Isabelle Ribeiro Barbosa

**Affiliations:** I Universidade Federal do Rio Grande do Norte Centro de Ciências da Saúde Departamento de Saúde Coletiva Natal RN Brasil Universidade Federal do Rio Grande do Norte . Centro de Ciências da Saúde . Departamento de Saúde Coletiva . Natal , RN , Brasil

**Keywords:** Rural Population, Mental Disorders, epidemiology, Risk Factors, Socioeconomic Factors

## Abstract

**OBJECTIVE:**

To identify the prevalence and factors associated with common mental disorders among farmers living in a medium-sized municipality in Northeastern Brazil between 2019 and 2020.

**METHODS:**

Trained interviewers applied the standardized questionnaire in 450 participants. Sociodemographic, health, income and working characteristics were assessed. The screening of common mental disorders was performed using the Self-Reporting Questionaire, with the cutoff point ≥ 7 for women and ≥ 5 for men. Poisson regression with robust estimation was applied to verify the prevalence ratios in the bivariate and multivariate analysis.

**RESULTS:**

The prevalence of common mental disorders among farmers was 55.1% (95%CI: 50.4–59.6). The variables that remained significant and associated with common mental disorders were: men (PR = 1.7), > 60 years old (PR = 0.5), poor or very poor self-assessment of health (PR = 1.4), previous mental health treatment (PR = 1.2), alcohol abuse (PR = 1.2) and loss of production (PR = 1.3).

**CONCLUSION:**

These results indicate that common mental disorders are associated with individual factors and with the farmers’ context of life and work, which shows the importance of social, economic and health services support to this group of workers.

## INTRODUCTION

Common mental disorders (CMD) comprise the presence of different symptoms for at least seven days, especially irritation, fatigue, anxiety, difficulty to concentrate and sleep disorders ^1^ , which can cause significant functional disability with psychosocial impairments and physical complaints ^2^ . Besides, CMDs are considered one of the most prevalent psychic morbidities worldwide ^3^ .

CMDs are associated with poor prognosis of some morbidities, high effect on health costs and economic productivity ^4^ . The most affected groups are young people, women, and single people. The main risk factors for these disorders are low socioeconomic status, psychological diseases, poor physical and reproductive health, and gender disadvantage ^5^ , representing a great public health problem due to its high prevalence and severe effects on personal and family well-being, work and use of health care services ^6^ .

CMDs are considered the most prevalent mental suffering worldwide and it is predicted that by 2030 they will be among the major disabling causes ^7^ . A systematic review with metanalysis, which analyzed 174 cross-sectional studies from developed and developing countries, showed that one out of five adults (17.6%) experienced a common mental disorder in the previous 12 months and 29.2% had this experience during life ^8^ . In Brazil, recent estimates have shown that CMDs are, respectively, the fifth and sixth causes of years of life with disability ^9^ . The prevalence of CMD ranges from 20% to 56% in the Brazilian adult population, affecting mainly women and workers ^2^ . The prevalence of these disorders ranges from 23.3% to 66.9% in rural areas ^6^ .

Regarding farmers and rural populations, the difficulty to access health care services and the high cost of psychiatric treatments contribute to some mental health-related care being neglected ^10^ . Besides, stressful characteristics of the work environment, such as long distances, isolation, difficulty to develop another work activity, the decline of the economy, irregular income and exposure to pesticides may be associated with the development of these disorders ^11^ . More specifically, studies have analyzed the association between the prevalence of CMD and the socioeconomic conditions of certain human groups ^12^ .

The Brazilian rural population has as historical mark a complex picture of inequalities and inaccessibility to many public policies. The lack of infrastructure, the typical problems of lack of social development, the high poverty rates, poor working conditions and education, affect the rural population’s mental health. However, few studies address the theme on a national and international scale ^13^ .

The municipality of Caicó is in the Seridó region of the state of Rio Grande do Norte (RN), which is a region naturally susceptible to the arid weather, with periodic droughts, irregular and sparse rains, water deficiency, besides the presence of desertification and salinization processes. Family farmers from Seridó face a situation of socioeconomic and environmental vulnerability due to historical processes of exclusion of family farming in Northeastern Brazil and the state of Rio Grande do Norte, associated with strict environmental conditions, typical of the semi-arid area ^14^ . Mental health issues require special attention in this municipality because, according to a study, the municipality of Caicó had a suicide rate of 15.8/100,000 inhabitants between 2005 and 2007, occupying the third place among the 20 Brazilian cities with at least 50,000 inhabitants with the highest suicide coefficients ^15^ .

Considering the situation of exclusion experienced by family farming workers, studies that offer relevant data to implement prevention strategies at primary and secondary levels are needed, as well as health promotion to help the planning of interventions and healthy practices focused on mental health in rural communities.

We aimed to estimate the prevalence and factors associated with CMD in farmers in the municipality of Caicó-RN to contribute to the advancement of mental health research in rural populations, specifically among farmers.

## METHODS

This is a cross-sectional study, with data collected from August 2019 to March 2020, from farmers in the municipality of Caicó-RN. The municipality of Caicó is in the micro-region of Seridó, in the Central Potiguar mesoregion, 283 km from the state capital of Rio Grande do Norte. The estimated population for 2019 is 67,952 people and the population density is 55.31 inhabitants/km ^2^ . It has a Human Development Index of 0.710, a predominance of the Caatinga biome and its main economic activities are livestock, family farming and services ^17^ ( Figure ).

**Figure:**
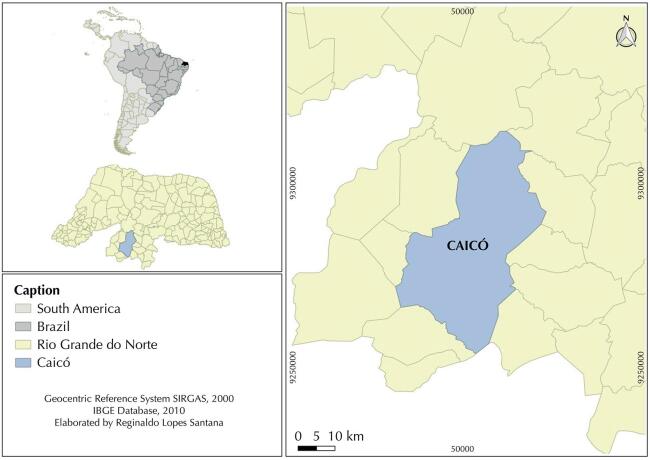
Geographical location of the municipality of Caicó, state of Rio Grande do Norte (RN).

The studied population consisted of farmers registered in the Rural Workers Union of the municipality of Caicó-RN. The inclusion criteria for this study were: being registered in the union and aged between 18 and 80 years old, which constituted a population of 2,000 people.

A 19.7% prevalence of CMD was considered to estimate the sample size for finite populations ^18^ . Considering 4% absolute margin of error, 30% non-response rate, 19.7% estimated proportion of the event and finite population of 2,000 farmers, the estimated sample represented 450 farmers. The participants were allocated by a simple random sampling in which all elements of the population were included (2,000 individuals). The interviews, conducted by previously trained interviewers, occurred at home, in a reserved place, after the participant’s consent.

The dependent variable was the presence of CMD, analyzed by the SRQ-20 questionnaire (Self-Reporting Questionaire), which was originally developed in 1980 ^18^ and validated over the years in many samples of the Brazilian population by many researchers, who reported 83% sensitivity and 80% specificity of this instrument ^19^ . The questionnaire addresses the presence of physical and psychic symptoms in the last 30 days. In this study, cutoff points ≥ 5 for men and ≥ 7 for women were used to classify the presence of CMD ^22 , 23^ .

The independent variables were grouped into three categories: (1) sociodemographic: gender (men; women); age group in years (18–39, 40–59 and ≥ 60); race/skin color (White and other, Mixed-race, Black); marital status (married; single/divorced; widower/widow); religious (yes; no); number of residents in the household (0–2; 3–4; ≥ 5); access to sanitation - garbage collection and water from the public network (yes; no); schooling (without schooling, up to elementary school, up to high school, up to higher education); and area of residence (urban area; rural area). (2) health aspects: self-assessment of health (very good/good; regular; bad/very bad); diagnosis of mental disorders in the family (yes; no); have already undergone mental health treatment (yes; no); smoking habit (yes; no); alcohol abuse by the CAGE scale (Cut down, Annoyed by criticism, Guilty and Eye-opener) (yes; no); drug use (yes; no); if sought and managed to get health care in the last 12 months (yes; no); if the family is assisted by the community health agent (yes; no). (3) income and work: employed (yes; no); individual monthly income (no income; less than a minimum wage; one minimum wage; above one minimum wage); agrarian indebtedness with banks, relatives or loan sharks (yes; no); access to a government credit program for agriculture (yes; no); partially or totally lost production in the last two years (yes; no); have contact with pesticides (yes; no); use personal protective equipment (PPE) when handling pesticides (yes; no); needed hospital care due to poisoning (yes; no); number of working hours per day (< 6h; > 6h).

These data were collected from an adapted version of the sociodemographic-environmental questionnaire elaborated by the laboratory of strategic analysis of the Geology department of the Universidade Federal do Rio Grande do Norte (UFRN) ^24^ .

Alcohol abuse was analyzed by the CAGE questionnaire. This questionnaire consists of four questions: (1) Have you ever felt that you should decrease the amount of drink or stop drinking? (2) Do people annoy you because they criticize your way of drinking? (3) Do you feel guilty about the way you usually drink? (4) Do you usually drink in the morning to reduce nervousness or hangover? Alcohol abuse was confirmed in case of affirmative answer to at least two questions in the questionnaire.

A descriptive analysis of the studied participants was performed by absolute and relative frequencies. The chi-square test was applied to compare the proportions of the outcome between the categories of each variable. Poisson regression with robust variance was used to analyze the associated factors and estimate prevalence ratios (PR). The multiple analysis was constructed based on the set of variables that showed a p value < 0.20 in the bivariate analysis. The blocks of characteristics of the variables were adopted as entry criterion in the model, first the variables of sociodemographic aspects, followed by health characteristics and finally those of income and work. The final model was composed of the variables that remained significant in the model (p < 0.05). The data were analyzed using the Stata 13 statistical package (StataCorp LP, College Station, USA), with 5% significance level.

This study was approved by the research ethics committee of Hospital Universitário Onofre Lopes (HUOP), Universidade Federal do Rio Grande do Norte (UFRN), under the registration CAAE 15532919.5.0000.5292, on July 5, 2019, and is aligned with the guidelines for research in human beings in Brazil, according to Resolution 466 of December 2012. All participants signed the informed consent form before the interviews.

## RESULTS

Out of 450 farmers included in the study, 58.9% are men, 52.8% declared themselves as Black (Black and Mixed-race), 41.3% are in the age group of 40–59 years old, 86% have up to elementary school, 76% do not have access to basic sanitation, 52% evaluate their health as good or very good, 94.7% are covered by the *Estratégia Saúde da Família* (ESF - Family Health Strategy), 94% have an income of up to one monthly minimum wage, 47.7% have access to credit, 43.7% have debts, 59.7% have already lost production and 58.67% have no contact with pesticides. The prevalence of smoking was 27% and alcohol abuse was 32%.

The prevalence of CMD among farmers was 55.1% (95%CI: 50.4–59.6). The bivariate analysis showed an association between the outcome and men (PR = 1.7; 95%CI: 1.3–2.2), > 60 years old (PR = 0.7; 95%CI: 0.4–0.9), alcohol abuse (PR = 1.6; 95%CI: 1.3–2.1), having a diagnosis of mental disorder in the family (PR = 1.4; 95%CI 1.0–1.8), not having access to credit (PR = 0.7; 95%CI: 0.5–0.9) and have contact with pesticides (PR = 1.3; 95%CI: 1.0–1.7) (Tables 1, 2 and 3).

**Table 1:** Descriptive and bivariate analysis of the presence of common mental disorders and their association with sociodemographic aspects of farmers in the municipality of Caicó-RN.

Variable	n (%)	Common mental disorder			Prevalence ratio		
		%	95%CI	p^a^	_crude_PR	95%CI	p^b^
Gender							
Women	185 (41.1)	38.3	31.6–45.6	< 0.001^c^	1	-	-
Men	265 (58.9)	66.7	60.8–72.2		1.7	1.3–2.2	< 0.005^c^
Age							
18–39 years	139 (31.0)	61.1	52.7–68.9	0.003^c^	1	-	-
40–59 years	186 (41.3)	42.1	33.9–51.2		0.9	0.7–1.2	0.817
≥ 60 years	125 (27.7)	59.1	51.8–66.0		0.7	0.4–0.9	0.036^c^
Marital status							
Married	315 (70.0)	56.1	50.6–61.5	0.669	1	-	-
Single/divorced	100 (22.3)	54.0	44.1–63.5		0.9	0.7–1.3	0.798
Widow/widower	35 (7.7)	48.5	32.4–64.9		0.8	0.5–1.4	0.566
Race/Skin color							
White/other	212 (47.2)	49.5	42.8–56.2	0.080	1	-	-
Black	42 (9.3)	59.5	44.0–73.3		1.2	0.7–1.8	0.409
Mixed-race	196 (43.5)	60.2	53.1–66.8		1.2	0.9–1.5	0.146
Schooling level							
Higher education	38 (8.5)	57.8	44.1–74.8	0.135	1	-	-
High school	21 (4.7)	52.4	31.3–72.6		0.8	0.3–1.7	0.608
Elementary school	256 (56.8)	51.1	57.1–68.9		0.8	0.5–1.3	0.592
No schooling	135 (30.0)	47.6	66.7–81.4		1.0	0.6–1.7	0.726
Religion							
Yes	332 (73.7)	57.2	51.8–62.4	0.130	1	-	-
No	118 (26.3)	49.1	41.8–59.8		0.8	0.6–1.1	0.311
Number of residents							
0–2 individuals	101 (22.4)	54.4	44.6–57.2	0.073	1.0	0.7–1.4	0.662
3–4 individuals	221 (49.2)	50.6	44.0–63.9		1	-	-
≥ 5 individuals	128 (28.4)	36.7	54.5–71.2		1.2	0.9–1.6	0.128
Area of residence							
Urban area	34 (7.5)	52.9	36.1–69.0	0.791	1	-	-
Rural area	416 (92.5)	55.2	50.4–60.0		0.7	0.5–1.1	0.223
Sanitation access							
Yes	108 (24.0)	56.4	46.9–65.5	0.743	1	-	-
No	342 (76.0)	54.6	49.3–59.9		0.9	0.7–1.2	0.826

95%CI: 95% confidence interval; _crude_PR: crude prevalence ratio.

^a^ Statistical significance obtained by the chi-square test.

^b^ Statistical significance obtained by the Poisson regression.

^c^ Significant value at 5%.

**Table 2:** Descriptive and bivariate analysis of the presence of common mental disorders and their association with health aspects among farmers in the municipality of Caicó-RN.

Variables	n (%)	Common mental disorder			Prevalence Ratio		
		%	95%CI	p ^a^	_crude_ PR	95%CI	p ^b^
Self-assessment of health							
Very good/good	234 (52.0)	51.7	45.2–58.0	0.052	1	-	-
Regular	129 (29.7)	53.4	44.8–61.9		1.0	0.7–1.3	0.823
Bad/very bad	87 (19.3)	66.6	56.0–75.8		1.2	0.9–1.7	0.112
Diagnosis of mental disorder in the family							
No	200 (44.4)	45.0	38.2–51.9	< 0.005 ^c^	1	-	-
Yes	250 (55.6)	63.2	57.0–68.9		1.4	1.08–1.8	0.010 ^c^
Have already undergone mental health treatment							
No	332 (71.6)	52.2	46.9–57.6	0.047 ^c^	1	-	-
Yes	118 (28.4)	62.5	53.7–70.4		1.2	0.9–1.5	0.184
Smoking habit							
No	327 (72.7)	51.6	46.2–57.0	0.017 ^c^	1	-	-
Yes	123 (27.3)	64.2	55.3–72.2		1.2	0.9–1.6	0.111
Drug use							
No	445 (98.9)	54.8	50.1–59.4	0.261	1	-	-
Yes	5 (1.1)	80.0	25.53–97.90		1.4	0.5–3.9	0.454
Alcohol abuse							
No	306 (68.0)	45.4	39.9–51.0	< 0.005 ^c^	1	-	-
Yes	144 (32.0)	75.6	67.9–82.0		1.6	1.3–2.1	< 0.005 ^c^
Access to health care services							
Yes	340 (75.6)	57.9	52.6–63.1	0.034 ^c^	1	-	-
No	110 (24.4)	46.3	37.2–55.7		0.8	0.5–1.0	0.156
FHS coverage							
Yes	426 (94.7)	55.1	50.3–59.8	0.924	1	-	-
No	24 (5.3)	54.1	34.1–72.8		0.98	0.5–1.7	0.949

95%CI: 95% confidence interval; _crude_ PR: crude prevalence ratio; FHS: Family Health Strategy.

^a^ Statistical significance obtained by the chi-square test.

^b^ Statistical significance obtained by the Poisson regression.

^c^ Significant value at 5%.

**Table 3:** Descriptive and bivariate analysis of the presence of common mental disorders and their association with income and work aspects of farmers in the municipality of Caicó-RN.

Variable	n (%)	Common mental disorder			Prevalence ratio		
		%	95%CI	p ^a^	_crude_ PR	95%CI	p ^b^
Employed							
Yes	313 (69.6)	60.0	54.5–65.3	0.001 ^c^	1	-	-
No	137 (30.4)	43.7	35.6–52.2		0.7	0.5–0.9	0.033 ^c^
Monthly income							
No income	30 (6.7)	50.0	32.5–67.4	0.727	0.84	0.4–1.7	0.636
Up to 1/2 minimum wage	81 (18.0)	50.6	44.5–66.0		0.93	0.5–1.6	0.825
1 minimum wage	312 (69.3)	55.5	65.5–75.6		1.19	0.7–1.9	0.491
> 1 minimum wage	27 (6.0)	56.7	39.8–76.1		1	-	-
Hours of daily work							
< 6 hours	302 (67.1)	55.6	49.9–61.1	0.838	1	-	-
> 6 hours	111 (24.7)	56.7	47.3–65.7		1.0	0.7–1.36	0.892
Not applicable	37 (8.2)	-	-				
Access to credit							
Yes	215 (47.8)	63.2	56.5–69.4	0.002 ^c^	1	-	-
No	217 (48.2)	48.3	44.7–55.0		0.7	0.5–0.9	0.039 ^c^
Not applicable	18 (4.0)	-	-				
Have debts							
No	235 (52.2)	49.3	42.9–55.7	0.003 ^c^	1	-	-
Yes	197 (43.8)	63.4	56.4–69.9		1.2	0.9–1.6	0.051
Not applicable	18 (4.0)	-	-				
Relationship with the land							
Owner	307 (68.2)	55.0	49.4–60.5	0.710	1	-	-
Tenant	84 (18.7)	53.5	42.8–64.0		0.9	0.7–1.35	0.695
Employee/temporary	46 (10.2)	60.8	54.7–81.2		1.1	0.1–1.6	0.770
Not applicable	13 (2.9)	-	-				
Already had loss of production							
No	149 (33.1)	48.3	46.9–62.8	0.028 ^c^	1	-	-
Yes	269 (59.8)	59.4	66.0–76.8		1.2	0.9–1.6	0.143
Not applicable	32 (7.1)	-	-				
Have contact with pesticides							
No	264 (58.7)	50.3	44.3–56.4	0.002 ^c^	1	-	-
Yes	146 (32.4)	66.4	58.3–73.6		1.3	1.0–1.7	0.038 ^c^
Not applicable	40 (8.9)	-	-				
Use PPE							
Yes	84 (18.7)	60.7	49.8–70.6	0.671	1	-	-
No	200 (44.4)	58.0	51.0–64.6		0.9	0.6–1.3	0.785
Not applicable	166 (36.9)	-	-				
Pesticide poisoning							
No	294 (65.3)	58.1	52.4–63.7	0.078	1	-	-
Yes	22 (4.9)	77.2	54.9–90.4		1.3	0.8–2.1	0.264
Not applicable	134 (29.8)	-	-				

95%CI: 95% confidence interval; _crude_ PR: crude prevalence ratio; PPE: personal protective equipment.

^a^ Statistical significance obtained by the chi-square test.

^b^ Statistical significance obtained by the Poisson regression.

^c^ Significant value at 5%.

Some variables with p < 0.200 in the bivariate analysis were tested in the multivariate model: number of residents in the household, having undergone mental health treatment, smoking habit, access to health care services, having debts and loss of production.

In the multivariate model, the variables that remained significant and associated with CMD were: men (PR = 1.7), > 60 years old (PR = 0.5), having poor or very poor self-assessment of health (PR = 1.4), previous mental health treatment (PR = 1.2), alcohol abuse (PR = 1.2) and having had loss of production (PR = 1.3) (Table 04).

**Table 4:** Multivariate model for common mental disorders and their association with sociodemographic, health and work aspects of farmers in the municipality of Caicó-RN.

Variables	_adjusted_ PR	95%CI	p ^a^
Gender			
Women	1	-	-
Men	1.7	1.3–2.1	< 0.005
Age			
18–39 years	1	-	-
40–59 years	0.9	0.7–1.1	0.497
≥ 60 years	0.5	0.4–0.7	< 0.005
Self-assessment of health			
Very good/good	1	-	-
Regular	1.1	0.9–1.4	0.168
Bad/very bad	1.4	1.1–1.7	< 0.005
Have already undergone mental health treatment			
No	1	-	-
Yes	1.2	1.0–1.5	0.004
Alcohol abuse			
No	1	-	-
Yes	1.2	1.0–1.5	0.004
Already had loss of production			
No	1	-	-
Yes	1.3	1.0–1.5	0.004
Constant	0.27	0.2–0.3	< 0.005

95%CI: 95% confidence interval; _adjusted_ PR: adjusted prevalence ratio.

^a^ Statistical significance obtained by the Poisson regression.

## DISCUSSION

Our study identified a high prevalence of CMD in farmers in the municipality of Caicó, associated with sociodemographic, work and health factors, such as gender, age group, self-assessment of health status, alcohol abuse, mental health treatment and loss of agricultural production. The studied population is mostly male, people > 40 years old, married, with low schooling, low income, no access to basic sanitation and with prevalence of alcohol abuse > 30%. The interviews indicated that most of these farmers have difficulties to obtain treated water, health care services, access to rural credit and land ownership, besides being in debt and with a history of loss of agricultural production.

We emphasize the low schooling level in the studied population, since 86.89% of the subjects have up to elementary school. The low schooling profile observed among workers is similar to the profile of farmers found in studies conducted in other Brazilian states ^25 , 26^ . Although our study did not show association between CMD and schooling, we emphasize that schooling is directly related with mental health because it influences life choices, aspirations, opportunities, self-esteem and acquisition of new knowledge, which can contribute to healthier attitudes and behaviors ^27^ .

Socioeconomic variables such as poverty and precarious working conditions, which are characteristics of most rural contexts in the Northeast, tend to contribute to the higher risk of developing CMD among individuals living and working in rural areas, showing the need for greater attention and care to this population’s mental health ^25^ . The social vulnerability of rural families in the municipality of Caicó is considered moderate, but economic and technological vulnerabilities are considered very high ^28^ .

Our study showed a higher prevalence of CMD than that found in other rural locations, such as the study conducted with older adults living in rural areas of the municipality of Jequié-BA (47.4%) ^26^ , women from rural areas of the municipality of Rio Grande-RS (36.4%) ^27^ and rural communities of Atibaia-SP (23.36%) ^29^ . In individuals living in rural communities in other countries, the prevalence of CMD assessed by the SRQ-20 was 27.2% in Southeastern Ethiopia ^5^ and 22.8% in women from rural areas in India ^30^ .

Although these studies used the SRQ-20 as a diagnostic instrument, the cutoff point adopted to detect CMD differ in the studies of different researchers, which may partially explain the differences observed in the prevalence of mental disorders between countries and within the same country. The researchers compared the results with studies in urban areas due to the lack of validation of the SRQ-20 for the rural population in Brazil, adopting the same cutoff score and methodology ^28^ . Besides, the differences in the time of evaluation of symptoms and the characteristics of each rural population under study may contribute to this variability ^6^ . The application of the SRQ-20 has shown good performance in specific populations, such as rural populations ^5 , 26 , 27 , 29 , 30^ .

Regarding gender, the prevalence of CMD was higher among men, which differs from the literature. Overall, women are more affected by CMD, and the factors associated with this higher prevalence are > 50 years old, separated/divorced, with low schooling and income ^30^ . However, due to the strong relationship that men have with work, any failure can affect their social and personal context, generating emotional/psychological problems ^31^ .

The variable of access to health care services did not show statistical significance in our final model, but we emphasize the male representations of health-illness, care and the search for health care. The high prevalence of CMD in men may be related to the barriers to the male presence in health care services, which connects to the gender identity structure (the notion of invulnerability, the search for risk as a value), thus, hindering the verbalization of their health needs during care ^32^ .

In rural cities, especially in the Northeast region of Brazil, there are few services directed to mental health care and less resources to diagnose and treat mental disorders. For lighter cases, treatment is usually limited to medicine care or referral to specialists, which shows the deficiencies in the local mental health care network and the difficulties of primary health care providers to deal with these forms of suffering ^25^ .

Despite the Brazilian Family Health Strategy innovations regarding structure and work process when compared to primary care and conventional health, it has not yet been able to transform mental health care, such as greater and better availability of professionals and resources, the structuring of own flows for mental health, psychosocial listening, the reception of otherness and integrated networking ^33^ .

In our study, the presence of CMD was associated with the previous use of mental health care services. Studies show that individuals who live in rural areas and who have mental disorders usually claim not to seek health care services due to geographic inaccessibility and unsatisfactory reception experiences ^12 , 34^ . However, the high prevalence of mental disorders such as alcohol abuse ^35^ and suicidal behavior ^36^ in populations living in rural areas may explain the greater demand for these services.

We emphasize that the health network of the municipality of Caicó consists of services of different levels of complexity of health care. In the field of mental health, the municipality has a Psychosocial Care Center III, a Psychosocial Care Center Alcohol and Drugs, and Therapeutic Residences ^37^ . The higher prevalence of previous mental health-related treatments among those with CMD may be an indicator of mental health care provision.

Regarding self-assessment of health status, studies ^30 , 38^ show that individuals with CMD have a low assessment of their own health, which corroborates our results. Overall, these individuals have other comorbidities associated with CMD, which can cause loss of quality of life and influence the self-assessment of their health status ^39^ .

The prevalence of CMD was lower among individuals > 60 years old. This finding differs from other authors ^6^ , who found higher prevalence of CMD among older adults, with low schooling level and smokers. International studies in the general population show prevalence of CMD in older adults ranging from 32.4% in Ethiopia ^40^ to 51.8% in Denmark ^41^ . In Brazil, the study conducted in Ibicuí-BA showed a 55.8% prevalence of mental disorders in older adults ^6^ and 29.7% in Campinas-SP ^42^ . There is an increasing gradient of CMD as age increases ^38^ , because many factors can contribute to this increase in the aging process, including the high presence of morbidities and disabilities, poor living conditions, stressful life events, social isolation and economic difficulties.

Although race and skin color were not associated with the outcome in the multivariate analysis, in cultural and social terms, Black people are still linked to vulnerabilities and the worst economic, schooling, working and housing conditions ^43^ . Besides, studies show racial disparities in access to treatment of mental disorders, in which the Black population has less access to mental health services ^44 , 45^ . We emphasize how ethnic-racial issues are scarcely addressed in health studies and few discussions on how discrimination and oppression affect mental health ^46^ .

Loss of production was a factor associated with CMD in our study. A study ^47^ identified that losses in production due to climate or pests reduce crops by up to 40% worldwide, especially rice (from 25% to 41%), corn (from 20% to 41%), potatoes (from 8% to 21%) and soybeans (from 11% to 32%).

Climate is the main factor responsible for oscillations and frustrations of agricultural crops in Brazil. This dependence on rain conditions affects the Northeast region, in its semi-arid area, in which the municipality of Caicó is located. This piece of the Northeastern territory is formed by a set of spaces that are characterized by a negative water balance, caused by the mean annual precipitations < 800 mm, mean insolation of 2,800h/year, mean annual temperatures ranging from 23°C to 27°C, evaporation of 2,000mm/year and mean relative humidity of the air around 50% ^48^ .

In the Seridó region, the naturally difficult climatic conditions and the anthropic pressures such as extensive livestock farming, dry farming (practiced in the rainy months in the region) with inadequate cropping and techniques, extractive ceramics and mining industry result in degraded areas and reduce farmers’ adaptation to future climate change, which increases their vulnerability. All these factors negatively affect family agricultural production already weakened in this region ^49^ .

In Northeastern municipalities, farmers enrolled in the *Programa Nacional de Fortalecimento da Agricultura Familiar* (PRONAF - National Program for Strengthening Family Agriculture) and who fit in the PRONAF-B credit line (annual income of up to R$ 20,000.00) have access to a credit program financed by the Banco do Nordeste of Brazil since 2005, the *Programa de Microcrédito Rural Orientado e Acompanhado* (Oriented and Accompanied Rural Microcredit Program), called Agroamigo ^50^ .

The producers’ financial difficulties increase in case of loss of production without the expected profit from that crop, being unable to pay off debts, bank loans or support their family, factors that directly affect the mental health of rural workers. Financial debts are related to many health problems, particularly mental health ^51^ . In Caicó-RN, financing is only available for a part of the rural families despite most of them survive from agriculture, since most of these families lack agricultural implements for management and commercialize their production in agro-industries, directly to consumers and retailers ^29^ .

The land structure of Seridó is based on the great area destined to large properties, distributed to few buildings. The Eastern and Western Seridó of Rio Grande do Norte have the greatest number of large properties and municipalities with concentrated land structure, especially the municipality of Caicó ^52^ . There are 9,970 family farming establishments in the Seridó region, which represents 80.6% of the agricultural properties in the region. However, these properties occupy only 24.2% of the total agricultural area ^14^ .

Associated with land concentration, the intense process of desertification that the Northeastern semi-arid is going through leads to soil degradation, reduction of agricultural and cattle productive capacity, reduction of agricultural and cattle income, and deterioration of the social conditions of the local population ^52^ . Factors such as the reduced size of properties, lack of resources and technical assistance, and low schooling level limit the resilience of family farming. Thus, agricultural production is affected during droughts or floods. Farmers’ adaptation alternatives include: reduction of agricultural production, migration of young people to urban areas of the state and obtaining income off the property ^14^ .

The Country’s social indicators have improved in recent decades, especially in rural areas, such as the decrease in inequality, poverty and extreme poverty rates, increase in the *per capita* income and in housing, schooling, health and leisure. However, the situation is still alarming since, for example, one out of four Brazilian people living in the countryside is in a situation of extreme poverty ^12^ .

Regarding alcohol intake, alcohol abuse was associated with a higher prevalence of CMD. Moderate alcohol consumption can have a positive psychological effect, relieving emotional distress and effective to deal with social and anxiety situations ^53^ . However, mental disorders and alcohol abuse have a greater negative effect on more socioeconomically disadvantaged populations due to their situations of vulnerability and few health resources. In realities involving rural settlement areas in Brazil, the rural population is more vulnerable to conditions susceptible to develop CMDs and diseases related to alcohol abuse due to the harsh living and working conditions, poverty, social isolation and their low coverage of public services ^54^ .

Alcohol abuse is associated with an increase in the mortality and morbidity rate due to its potential to cause diseases and is related to loss of quality of life ^55^ . A study ^6^ showed that the prevalence of CMD in older adults was higher among those who regularly consumed alcoholic beverages (61.5%), who were currently smoking (58.3%) and among those who had already smoked at some point in their lives (56.1%).

Although our study did not find a relationship between mental disorders and smoking habits, other studies indicate an association between them based on the action of tobacco on the neurotransmitter system, which influences the psychopathological condition and causes reactions, such as: emotional symptoms, behavior problems and relationship problems ^54^ . A longitudinal study conducted in India with 2,094 women aged between 18 and 45 years showed that smokers had an incidence of mental disorders four times higher than non-smokers ^55^ . Besides, nicotine acts on the dysregulation in the hypothalamic-pituitary-adrenal axis and increases the action of cortisol in the brain, a change that is characteristic of depressive symptoms, therefore, smoking could cause greater susceptibility to develop CMD ^27^ .

Our results must be cautiously evaluated due to the possible limitations of the cross-sectional study. Selection bias may have occurred, since the selected workers had to be affiliated with the local union at the time of the research. The instrument used in data collection, the SRQ-20, is another limitation, which was not self-applied. Most participants had difficulties to understand the questions asked, probably due to low schooling, forcing a greater participation of the researchers. These interventions may have caused information biases.

Our study suggests that CMDs are associated with the variables: gender, age group, self-assessment of health status, alcohol abuse, previous mental health treatment and loss of agricultural production. These findings may strengthen the discussion on the social determination of mental health, a debate that is still incipient among health teams and creators of health programs and policies. These results corroborate the literature, which shows that mental suffering among farmers and rural workers is a problem of great magnitude. Actions to improve living and working conditions can benefit the maintenance and strengthening of mental health conditions in this population.

In the context of the covid-19 pandemic, the population’s mental health conditions must be considered in face of the multiple reflexes that this pandemic has caused, significantly changing the mental health condition of the population worldwide ^56^ . Regarding the studied issue, these rural workers require special attention to their psychological demands that may worsen due to the pandemic, which urges the protection of the mental health of this vulnerable group.

Our findings emphasize the importance to strengthen the health care network, especially in psychosocial care services, aiming to develop preventive strategies for these groups and the decrease in mental illness rates. We concluded that the periodic monitoring of these farmers’ mental health is essential for early identification of problems that affect the workers’ health. Therefore, public administrators must propose strategies for monitoring and early intervention to minimize the symptoms and present manifestations, preventing the occurrence, emotional deterioration and psychological worsening.
