# Supplementary material for: Factors associated with common mental disorders among farmers in a medium-sized municipality in Northeastern Brazil
Source: Rev Saude Publica. 2022 Aug 2;56:74. doi: 10.11606/s1518-8787.2022056003522 (PMC9388066; doi:10.11606/s1518-8787.2022056003522)
Supplement: Translation

# Translation: Fatores associados aos transtornos mentais comuns entre agricultores em um município de médio porte no nordeste do Brasil

**Authors:** Roberta Machado Alves, Emelynne Gabrielly de Oliveira Santos, Isabelle Ribeiro Barbosa

**Affiliations:** I Brasil Universidade Federal do Rio Grande do Norte. Centro de Ciências da Saúde. Departamento de Saúde Coletiva. Natal, RN, Brasil

**Keywords:** População Rural, Transtornos Mentais, epidemiologia, Fatores de Risco, Fatores Socioeconômicos

## Abstract

**OBJETIVO:**

Identificar a prevalência e os fatores associados aos transtornos mentais comuns entre agricultores residentes em um município de médio porte no nordeste do Brasil, entre 2019 e 2020.

**MÉTODOS:**

Entrevistadoras treinadas aplicaram o questionário padronizado em 450 participantes. Investigou-se características sociodemográficas, de saúde, renda e trabalho. O rastreamento dos transtornos mentais comuns foi realizado mediante a utilização do questionário SRQ-20 ( *Self-Reporting Questionaire* ), com o ponto de corte ≥ 7 para mulheres e ≥ 5 para homens. Foi aplicada a regressão de Poisson com estimação robusta para verificar as razões de prevalência na análise bivariada e multivariada.

**RESULTADOS:**

A prevalência dos transtornos mentais comuns entre agricultores foi de 55,1% (IC95% 50,4–59,6). As variáveis que permaneceram significativas e associadas aos transtornos mentais comuns foram: ser do sexo masculino (RP = 1,7), ter mais de 60 anos (RP = 0,5), ter autoavaliação de saúde ruim ou muito ruim (RP = 1,4), ter realizado tratamento anterior para saúde mental (RP = 1,2), fazer uso abusivo do álcool (RP = 1,2) e ter tido perda de produção (RP = 1,3).

**CONCLUSÃO:**

Esses resultados indicam que os transtornos mentais comuns estão associados a fatores individuais e do contexto de vida e trabalho dos agricultores, o que demonstra a importância do suporte social, econômico e dos serviços de saúde a esse grupo de trabalhadores.

## INTRODUÇÃO

Os transtornos mentais comuns (TMC) compreendem a presença de diferentes sintomas por pelo menos sete dias, em destaque a irritação, fadiga, ansiedade, dificuldade de concentração e problemas em conciliar o sono ^1^ , podendo gerar incapacitação funcional significativa, com prejuízos psicossociais e queixas somáticas ^2^ . Além disso, os TMC são considerados uma das morbidades psíquicas mais prevalentes no mundo ^3^ .

Os TMC estão associados ao mau prognóstico de algumas morbidades, elevado impacto nos custos de saúde e produtividade econômica ^4^ . Na comunidade, os grupos mais afetados são jovens, mulheres e solteiros. Baixa condição socioeconômica, doenças psicológicas, saúde física e reprodutiva precária e desvantagem de gênero são os principais fatores de risco para esses transtornos ^5^ , representando, portanto, um importante problema de saúde pública devido à sua alta prevalência e graves efeitos sobre o bem-estar pessoal, familiar, trabalho e uso de serviços de saúde ^6^ .

Os TMC são considerados os sofrimentos mentais mais predominantes na população mundial e prevê-se que em 2030 estarão entre as maiores causas incapacitantes ^7^ . Em uma revisão sistemática, com metanálise, que analisou 174 estudos transversais de países desenvolvidos e em desenvolvimento, identificou-se que um em cada cinco adultos (17,6%) experimentou um transtorno mental comum nos 12 meses anteriores e 29,2% teve essa experiência ao longo da vida ^8^ . No Brasil, estimativas recentes mostraram que os TMC respondem, respectivamente, pelas quinta e sexta causas de anos de vida vividos com incapacidade ^9^ . Na população adulta brasileira, a prevalência de TMC varia de 20% a 56%, acometendo principalmente mulheres e trabalhadores ^2^ . Em áreas rurais, a prevalência desses transtornos varia de 23,3% a 66,9% ^6^ .

A respeito dos agricultores e das populações rurais, a dificuldade de acesso aos serviços de saúde e o elevado custo dos tratamentos psiquiátricos contribuem para que alguns cuidados relacionados à saúde mental sejam negligenciados ^10^ . Além disso, características estressoras do ambiente laboral, como as longas distâncias, o isolamento, a dificuldade em desenvolver outra atividade laboral, o declínio da economia, a renda irregular e a exposição a pesticidas podem estar associadas ao desenvolvimento desses transtornos ^11^ . De forma mais específica, os estudos têm analisado a associação entre a prevalência dos TMC e as condições socioeconômicas de determinados grupos humanos ^12^ .

A população rural brasileira tem como marca histórica um complexo quadro de desigualdades e dificuldades de acesso às mais diversas políticas públicas. A falta de infraestrutura e os problemas típicos da falta de desenvolvimento social, acompanhado dos altos índices de pobreza e miséria, precariedade das condições de trabalho e educação, trazem impactos à saúde mental da população do campo. Apesar de evidentes, são poucos os estudos que abordam a temática em escala nacional e internacional ^13^ .

O município de Caicó está localizado na região do Seridó do estado Rio Grande do Norte (RN), que é uma região naturalmente susceptível à aridez do clima, com ocorrência de secas periódicas, chuvas irregulares e esparsas, deficiência hídrica, além da presença dos processos de desertificação e salinização. Acredita-se que agricultores familiares seridoenses enfrentam uma situação de vulnerabilidade socioeconômica e ambiental, proveniente de processos históricos de exclusão da agricultura familiar no Nordeste brasileiro e no estado do Rio Grande do Norte, associada a condições ambientais rigorosas, características do Semiárido ^14^ . Nesse município, as questões de saúde mental merecem atenção especial, pois segundo um estudo o município de Caicó apresentou taxa de suicídio de 15,8/100 mil habitantes, entre os anos 2005 e 2007, ocupando o terceiro lugar entre as 20 cidades brasileiras com pelo menos 50 mil habitantes com maiores coeficientes de suicídio ^15 , 16^ .

Considerando a situação de exclusão vivida por trabalhadores da agricultura familiar, são necessários estudos que forneçam dados pertinentes para a implementação de estratégias de prevenção nos níveis primário e secundário, bem como promoção da saúde, visando auxiliar no planejamento de intervenções e práticas saudáveis voltadas para a saúde mental em comunidades rurais.

A fim de contribuir para o avanço das pesquisas em saúde mental em populações rurais, especificamente entre agricultores, este estudo tem por objetivo estimar a prevalência e os fatores associados aos TMC em agricultores do município de Caicó-RN.

## MÉTODOS

Trata-se de um estudo transversal, com dados coletados no período de agosto de 2019 a março de 2020, de agricultores do município de Caicó-RN. O município de Caicó está situado na microrregião do Seridó, na mesorregião Central Potiguar, a 283 km da capital do estado do Rio Grande do Norte. A população estimada para o ano de 2019 é de 67.952 pessoas e a densidade populacional de 55,31 habitantes/km ^2^ . Possui Índice de Desenvolvimento Humano de 0,710, predomínio do bioma caatinga e suas principais atividades econômicas são a pecuária, a agricultura familiar e os serviços ^17^ ( Figura ).

**Figura:** Localização geográfica do município de Caicó, estado do Rio Grande do Norte (RN).

A população do estudo foi composta por agricultores cadastrados no Sindicato dos Trabalhadores Rurais do município de Caicó-RN. Foram considerados critérios de inclusão para este estudo: ser cadastrado no sindicato e ter idade superior a 18 anos e inferior a 80 anos, o que constituiu uma população de duas mil pessoas.

Para o cálculo do tamanho da amostra para populações finitas, foi considerada a prevalência de TMC de 19,7% ^18^ . Considerando a margem de erro absoluta de 4%, taxa de não resposta de 30%, proporção estimada do evento de 19,7% e a população finita de dois mil agricultores, a amostra calculada correspondeu a 450 agricultores. A alocação dos participantes foi realizada por meio de uma amostragem aleatória simples em que todos os elementos da população foram incluídos (2.000 indivíduos). As entrevistas, realizadas por entrevistadores previamente treinados, ocorreram no domicílio em local reservado, após consentimento do participante.

A variável dependente foi a presença de TMC, analisada pelo questionário SRQ-20 ( *Self-Reporting Questionaire* ), que foi desenvolvido originalmente em 1980 ^18^ e validado ao longo dos anos em várias amostras da população brasileira, por diversos pesquisadores, que relataram sensibilidade de 83% e especificidade de 80% desse instrumento ^19^ . O questionário aborda a presença de sintomas físicos e psíquicos que podem ter incomodado nos últimos 30 dias. No presente estudo, para classificar a presença de TMC, foram utilizados os pontos de corte de ≥ 5 para os homens e ≥ 7 para as mulheres ^22 , 23^ .

As variáveis independentes foram agrupadas em três categorias, a saber: (1) sociodemográficas: sexo (masculino; feminino); faixa etária em anos (18–39, 40–59 e ≥ 60); raça/cor da pele (branca e outra, parda, preta); estado civil (casado; solteiro/divorciado; viúvo); possui religião (sim; não); número de residentes do domicílio (0–2; 3–4; ≥ 5); acesso a saneamento – coleta de lixo e água da rede pública (sim; não); escolaridade (sem escolaridade, até o ensino fundamental, até o ensino médio, até o ensino superior); e local de moradia (zona urbana; zona rural). (2) aspectos de saúde: autoavaliação de saúde (muito boa/boa; regular; ruim/muito ruim); diagnóstico de transtornos mentais na família (sim; não); já realizou tratamento para saúde mental (sim; não); tabagismo (sim; não); uso abusivo do álcool pela escala CAGE (do inglês *Cut down, Annoyed by criticism, Guilty e Eye-opener* ) (sim; não); uso de drogas (sim; não); se nos últimos 12 meses, procurou e conseguiu ter atendimento de saúde (sim; não); se a família é atendida pelo agente comunitário de saúde (sim; não). (3) renda e trabalho: está ocupado (sim; não); renda individual mensal (sem renda; menor que um salário mínimo; um salário mínimo; acima de um salário mínimo); possui algum endividamento agrário com bancos, parentes ou agiotas (sim; não); tem acesso a algum programa de crédito governamental para a agricultura (sim; não); nos últimos dois anos, perdeu, parcialmente ou totalmente a produção (sim; não); possui contato com agrotóxico (sim; não); durante a manipulação com o agrotóxico, utiliza equipamento de proteção individual (EPI) (sim; não); precisou de atendimento hospitalar por intoxicação (sim; não); número de horas diárias de trabalho (< 6h; > 6h).

Esses dados foram coletados a partir de uma versão adaptada do questionário sociodemográfico-ambiental elaborado pelo laboratório de análises estratégicas do departamento de Geologia da Universidade Federal do Rio Grande do Norte (UFRN) ^24^ .

O uso abusivo do álcool foi analisado pelo questionário CAGE. Esse questionário é composto por quatro perguntas: (1) Alguma vez o Sr.(a) sentiu que deveria diminuir a quantidade de bebida ou parar de beber? (2) As pessoas o(a) aborrecem porque criticam o seu modo de beber? (3) O Sr.(a) se sente culpado pela maneira com que costuma beber? (4) O Sr.(a) costuma beber pela manhã para diminuir o nervosismo ou a ressaca? Considerou-se o uso abusivo de álcool quando houve resposta afirmativa a pelo menos duas perguntas do questionário.

Foi realizada uma análise descritiva dos participantes do estudo pelas frequências absolutas e relativas. Foi aplicado o teste qui-quadrado para comparar as proporções do desfecho entre as categorias de cada variável. A regressão de Poisson com variância robusta foi usada para analisar os fatores associados e estimar as razões de prevalências (RP). A análise múltipla foi construída baseando-se no conjunto de variáveis que apresentaram um valor de p < 0,20 na análise bivariada. Adotamos como critério de entrada das variáveis no modelo os blocos de características das variáveis, sendo as primeiras as variáveis de aspectos sociodemográficos, seguidas das características de saúde e por último as de renda e trabalho. O modelo final foi composto apenas pelas variáveis que permaneceram significativas no modelo (p < 0,05). Os dados foram analisados usando o pacote estatístico Stata 13 (StataCorp LP, College Station, Estados Unidos), com nível de significância adotado de 5%.

Este estudo foi aprovado no comitê de ética em pesquisa do Hospital Universitário Onofre Lopes, da Universidade Federal do Rio Grande do Norte (CEP-HUOL-UFRN), sob o registro CAAE 15532919.5.0000.5292, em 5 de julho de 2019 e está em consonância com as diretrizes para pesquisas em seres humanos no Brasil, de acordo com a Resolução 466, de dezembro de 2012. Todos os participantes assinaram o Termo de Consentimento Livre e Esclarecido antes da condução das entrevistas.

## RESULTADOS

Dos 450 agricultores incluídos na pesquisa, 58,9% é do sexo masculino, 52,8% se declarou negro(a) (pretos e pardos), 41,3% estão na faixa etária de 40–59 anos, 86% tem até o ensino fundamental, 76% não possuem acesso a saneamento básico, 52% avaliam sua saúde como boa ou muito boa, 94,7% possuem cobertura da Estratégia Saúde da Família , 94% tem renda de até um salário mínimo mensal, 47,7% tem acesso a crédito, 43,7% possui dívidas, 59,7% já teve perda da produção e 58,67% não tem contato com agrotóxicos. A prevalência do tabagismo foi 27% e do uso abusivo de álcool foi de 32%.

A prevalência dos TMC entre agricultores foi de 55,1% (IC95% 50,4–59,6). A análise bivariada mostrou associação entre o desfecho e o sexo masculino (RP = 1,7; IC95% 1,3–2,2), ter acima de 60 anos (RP = 0,7; IC95% 0,4–0,9), fazer uso abusivo do álcool (RP = 1,6; IC95% 1,3–2,1), ter diagnóstico de transtorno mental na família (RP = 1,4; IC95% 1,0–1,8), não ter acesso a crédito (RP = 0,7; IC95% 0,5–0,9) e ter contato com agrotóxicos (RP = 1,3; IC95% 1,0–1,7) (Tabelas 1, 2 e 3).

**Tabela 1:** Análise descritiva e bivariada da presença de transtornos mentais comuns e sua associação com aspectos sociodemográficos de agricultores do município de Caicó-RN.

Variável	n (%)	Transtorno mental comum			Razão de prevalência		
		%	IC95%	p ^a^	RP _bruta_	IC95%	p ^b^
Sexo							
Feminino	185 (41,1)	38.3	31,6–45,6	< 0,001 ^c^	1	-	-
Masculino	265 (58,9)	66.7	60,8–72,2		1,7	1,3–2,2	< 0,005 ^c^
Idade							
18–39 anos	139 (31,0)	61,1	52,7–68,9	0,003 ^c^	1	-	-
40–59 anos	186 (41,3)	42,1	33,9–51,2		0,9	0,7–1,2	0,817
≥ 60 anos	125 (27,7)	59,1	51,8–66,0		0,7	0,4–0,9	0,036 ^c^
Estado civil							
Casado	315 (70,0)	56,1	50,6–61,5	0,669	1	-	-
Solteiro/divorciado	100 (22,3)	54,0	44,1–63,5		0,9	0,7–1,3	0,798
Viúvo	35 (7,7)	48,5	32,4–64,9		0,8	0,5–1,4	0,566
Cor da pele							
Brancos/outros	212 (47,2)	49,5	42,8–56,2	0,080	1	-	-
Pretos	42 (9,3)	59,5	44,0–73,3		1,2	0,7–1,8	0,409
Pardos	196 (43,5)	60,2	53,1–66,8		1,2	0,9–1,5	0,146
Escolaridade							
Até ensino Superior	38 (8,5)	57,8	44,1–74,8	0,135	1	-	-
Até ensino Médio	21 (4,7)	52,4	31,3–72,6		0,8	0,3–1,7	0,608
Ensino fundamental	256 (56,8)	51,1	57,1–68,9		0,8	0,5–1,3	0,592
Sem escolaridade	135 (30,0)	47,6	66,7–81,4		1,0	0,6–1,7	0,726
Possui religião							
Sim	332 (73,7)	57,2	51,8–62,4	0,130	1	-	-
Não	118 (26,3)	49,1	41,8–59,8		0,8	0,6–1,1	0,311
Número de residentes							
0–2 indivíduos	101 (22,4)	54,4	44,6–57,2	0,073	1,0	0,7–1,4	0,662
3–4 indivíduos	221 (49,2)	50,6	44,0–63,9		1	-	-
≥ 5 indivíduos	128 (28,4)	36,7	54,5–71,2		1,2	0,9–1,6	0,128
Local moradia							
Zona Urbana	34 (7,5)	52,9	36,1–69,0	0,791	1	-	-
Zona Rural	416 (92,5)	55,2	50,4–60,0		0,7	0,5–1,1	0,223
Acesso saneamento							
Sim	108 (24,0)	56,4	46,9–65,5	0,743	1	-	-
Não	342 (76,0)	54,6	49,3–59,9		0,9	0,7–1,2	0,826

IC95%: intervalo de confiança 95%; RP _bruta_: razão de prevalência bruta.

^a^ Significância estatística obtida pelo teste qui-quadrado.

^b^ Significância estatística obtida pela regressão de Poisson.

^c^ Valor significativo em nível de 5%.

**Tabela 2:** Análise descritiva e bivariada da presença de transtornos mentais comuns e sua associação com variáveis de aspectos de saúde entre agricultores do município de Caicó-RN.

Variáveis	n (%)	Transtorno mental comum			Razão de prevalência		
		%	IC95%	p ^a^	RP _bruta_	IC95%	p ^b^
Autoavaliação de saúde							
Muito boa/boa	234 (52,0)	51,7	45,2–58,0	0,052	1	-	-
Regular	129 (29,7)	53,4	44,8–61,9		1,0	0,7–1,3	0,823
Ruim/muito ruim	87 (19,3)	66,6	56,0–75,8		1,2	0,9–1,7	0,112
Diagnóstico de transtorno mental na família							
Não	200 (44,4)	45,0	38,2–51,9	< 0,005 ^c^	1	-	-
Sim	250 (55,6)	63,2	57,0–68,9		1,4	1,08–1,8	0,010 ^c^
Já realizou tratamento para saúde mental							
Não	332 (71,6)	52,2	46,9–57,6	0,047 ^c^	1	-	-
Sim	118 (28,4)	62,5	53,7–70,4		1,2	0,9–1,5	0,184
Tabagismo							
Não	327 (72,7)	51,6	46,2–57,0	0,017 ^c^	1	-	-
Sim	123 (27,3)	64,2	55,3–72,2		1,2	0,9–1,6	0,111
Uso de drogas							
Não	445 (98,9)	54,8	50,1–59,4	0,261	1	-	-
Sim	5 (1,1)	80,0	25,53–97,90		1,4	0,5–3,9	0,454
Uso abusivo do álcool							
Não	306 (68,0)	45,4	39,9–51,0	< 0,005 ^c^	1	-	-
Sim	144 (32,0)	75,6	67,9–82,0		1,6	1,3–2,1	< 0,005 ^c^
Acesso serviços de saúde							
Sim	340 (75,6)	57,9	52,6–63,1	0,034 ^c^	1	-	-
Não	110 (24,4)	46,3	37,2–55,7		0,8	0,5–1,0	0,156
Cobertura ESF							
Sim	426 (94,7)	55,1	50,3–59,8	0,924	1	-	-
Não	24 (5,3)	54,1	34,1–72,8		0,98	0,5–1,7	0,949

IC95%: intervalo de confiança de 95%; RP _bruta_: razão de prevalência bruta; ESF: Estratégia Saúde da Família.

^a^ Significância estatística obtida pelo teste qui-quadrado.

^b^ Significância estatística obtida pela regressão de Poisson.

^c^ Valor significativo em nível de 5%.

**Tabela 3:** Análise descritiva e bivariada da presença de transtornos mentais comuns e sua associação com aspectos de renda e trabalho de agricultores do município de Caicó-RN.

Variável	n (%)	Transtorno mental comum			Razão de prevalência		
		%	IC95%	p ^a^	RP _bruta_	IC95%	p ^b^
Ocupado(a)							
Sim	313 (69,6)	60,0	54,5–65,3	0,001 ^c^	1	-	-
Não	137 (30,4)	43,7	35,6–52,2		0,7	0,5–0,9	0,033 ^c^
Renda mensal							
Sem renda	30 (6,7)	50,0	32,5–67,4	0,727	0,84	0,4–1,7	0,636
Até 1/2 salário mínimo	81 (18,0)	50,6	44,5–66,0		0,93	0,5–1,6	0,825
1 salário mínimo	312 (69,3)	55,5	65,5–75,6		1,19	0,7–1,9	0,491
Acima de 1 salário mínimo	27 (6,0)	56,7	39,8–76,1		1	-	-
Horas de trabalho diária							
< 6 horas	302 (67,1)	55,6	49,9–61,1	0,838	1	-	-
> 6 horas	111 (24,7)	56,7	47,3–65,7		1,0	0,7–1,36	0,892
Não se aplica	37 (8,2)	-	-				
Tem acesso a crédito							
Sim	215 (47,8)	63,2	56,5–69,4	0,002 ^c^	1	-	-
Não	217 (48,2)	48,3	44,7–55,0		0,7	0,5–0,9	0,039 ^c^
Não se aplica	18 (4,0)	-	-				
Possui dívidas							
Não	235 (52,2)	49,3	42,9–55,7	0,003 ^c^	1	-	-
Sim	197 (43,8)	63,4	56,4–69,9		1,2	0,9–1,6	0,051
Não se aplica	18 (4,0)	-	-				
Relação com a terra							
Proprietário	307 (68,2)	55,0	49,4–60,5	0,710	1	-	-
Arrendatário	84 (18,7)	53,5	42,8–64,0		0,9	0,7–1,35	0,695
Assalariado/temporário	46 (10,2)	60,8	54,7–81,2		1,1	0,1–1,6	0,770
Não se aplica	13 (2,9)	-	-				
Já teve perda de produção							
Não	149 (33,1)	48,3	46,9–62,8	0,028 ^c^	1	-	-
Sim	269 (59,8)	59,4	66,0–76,8		1,2	0,9–1,6	0,143
Não se aplica	32 (7,1)	-	-				
Tem contato com agrotóxicos							
Não	264 (58,7)	50,3	44,3–56,4	0,002 ^c^	1	-	-
Sim	146 (32,4)	66,4	58,3–73,6		1,3	1,0–1,7	0,038 ^c^
Não se aplica	40 (8,9)	-	-				
Faz uso de EPI							
Sim	84 (18,7)	60,7	49,8–70,6	0,671	1	-	-
Não	200 (44,4)	58,0	51,0–64,6		0,9	0,6–1,3	0,785
Não se aplica	166 (36,9)	-	-				
Intoxicação por agrotóxicos							
Não	294 (65,3)	58,1	52,4–63,7	0,078	1	-	-
Sim	22 (4,9)	77,2	54,9–90,4		1,3	0,8–2,1	0,264
Não se aplica	134 (29,8)	-	-				

IC95%: intervalo de confiança de 95%; RP _bruta_: razão de prevalência bruta; EPI: equipamento de proteção individual.

^a^ Significância estatística obtida pelo teste qui-quadrado.

^b^ Significância estatística obtida pela regressão de Poisson.

^c^ Valor significativo em nível de 5%.

Algumas variáveis com p < 0,200 na análise bivariada foram testadas no modelo multivariado: número de residentes no domicílio, ter realizado tratamento para saúde mental, tabagismo, acesso a serviços de saúde, possuir dívidas e perda na produção.

No modelo multivariado, as variáveis que permaneceram significativas e associadas aos TMC foram: ser do sexo masculino (RP = 1,7), ter mais de 60 anos (RP = 0,5), ter autoavaliação de saúde ruim ou muito ruim (RP = 1,4), ter realizado tratamento anterior para saúde mental (RP = 1,2), fazer uso abusivo do álcool (RP = 1,2) e ter tido perda de produção (RP = 1,3) (Tabela 04).

**Tabela 4:** Modelo multivariado para os transtornos mentais comuns e sua associação com aspectos sociodemográficos, de saúde e de trabalho de agricultores do município de Caicó-RN.

Variáveis	RP _ajustada_	IC95%	p ^a^
Sexo			
Feminino	1	-	-
Masculino	1,7	1,3–2,1	< 0,005
Idade			
18–39 anos	1	-	-
40–59 anos	0,9	0,7–1,1	0,497
≥ 60 anos	0,5	0,4–0,7	< 0,005
Autoavaliação de saúde			
Muito boa/boa	1	-	-
Regular	1,1	0,9–1,4	0,168
Ruim/muito ruim	1,4	1,1–1,7	< 0,005
Já realizou tratamento para saúde mental			
Não	1	-	-
Sim	1,2	1,0–1,5	0,004
Uso abusivo do álcool			
Não	1	-	-
Sim	1,2	1,0–1,5	0,004
Já teve perda de produção			
Não	1	-	-
Sim	1,3	1,0–1,5	0,004
Constante	0,27	0,2–0,3	< 0,005

IC95%: intervalo de confiança de 95%; RP _ajustada_: razão de prevalência ajustada.

^a^ Significância estatística obtida pela regressão de Poisson.

## DISCUSSÃO

O presente estudo identificou elevada prevalência de TMC em agricultores no município de Caicó, associados a fatores sociodemográficos, laborais e de saúde, como o sexo, a faixa etária, a autoavaliação do estado de saúde, o uso abusivo de álcool, ter realizado tratamento para saúde mental e ter tido perda de produção agrícola. A população do estudo é de maioria masculina, de pessoas acima dos 40 anos, casados, com baixa escolaridade, baixa renda, sem acesso a saneamento básico e com prevalência de uso abusivo do álcool acima de 30%. Além disso, foi apontado nas entrevistas que a maioria desses agricultores apresenta dificuldades quanto à obtenção de água tratada, oferta de serviços de saúde, acesso a crédito rural e a propriedade de terras, além de estarem endividados e com histórico de perda da produção agrícola.

O baixo nível de escolaridade na população estudada merece destaque, uma vez que 86,89% dos sujeitos pesquisados possuem até o ensino fundamental. O perfil da baixa escolaridade observado entre os trabalhadores é similar ao perfil de agricultores encontrado nos estudos realizados em outros estados brasileiros ^25 , 26^ . Embora no presente estudo não tenha sido observada associação entre o TMC e a escolaridade, destacamos que a escolaridade possui uma relação direta com a saúde mental, pois influência nas escolhas de vida, nas aspirações, nas oportunidades, na autoestima e na aquisição de novos conhecimentos, os quais podem contribuir para atitudes e comportamentos mais saudáveis ^27^ .

As variáveis socioeconômicas como pobreza e condições precárias de trabalho, características da maioria dos contextos rurais do Nordeste, tendem a contribuir para o risco maior de desenvolvimento de TMC entre os indivíduos que vivem e trabalham nas áreas rurais, mostrando a necessidade de maior atenção e cuidado relacionados à saúde mental dessa população ^25^ . A vulnerabilidade social das famílias rurais do município de Caicó é considerada moderada, porém as vulnerabilidades econômica e tecnológica são consideradas muito altas ^28^ .

A prevalência de TMC encontrada neste estudo foi mais elevada do que a encontrada em outras localidades rurais, como no estudo realizado com idosos residentes na zona rural do município de Jequié-BA (47,4%) ^26^ , em mulheres da zona rural do município de Rio Grande-RS (36,4%) ^27^ e em comunidades rurais de Atibaia-SP (23,36%) ^29^ . Em indivíduos residentes em comunidades rurais de outros países, a prevalência de TMC rastreado pelo SRQ-20 foi de 27,2% no sudeste da Etiópia ^5^ e de 22,8% em mulheres de áreas rurais na Índia ^30^ .

Embora esses estudos tenham utilizado o SRQ-20 como instrumento diagnóstico, há diferença quanto ao ponto de corte adotado para detectar TMC nos estudos de diferentes pesquisadores, o que pode, ao menos em parte, explicar as diferenças observadas nas prevalências transtornos mentais entre os países e até mesmo dentro de um mesmo país. Devido à ausência de validação do SRQ-20 para população rural no Brasil, os pesquisadores comparam os resultados com os estudos em área urbana, adotando mesmo escore de corte e metodologia ^28^ . Além disso, sugere-se que as diferenças no tempo de avaliação dos sintomas e as características de cada população rural em estudo podem contribuir para essa variabilidade ^6^ . Mesmo assim, a aplicação do SRQ-20 tem demonstrado bom desempenho em populações específicas, como as populações rurais ^5 , 26 , 27 , 29 , 30^ .

Em relação ao gênero, houve maior prevalência de TMC entre os homens, o que não é consistente com os achados da literatura. Em geral, as mulheres são mais acometidas pelos TMC, para as quais ter idade acima dos 50 anos, ser separadas/divorciadas, com baixa escolaridade e renda são fatores associados à maior prevalência ^30^ . Todavia, considerando a forte relação que os homens têm com o trabalho, entende-se que qualquer falha ou insucesso pode afetar seu contexto social e pessoal, gerando problemas emocionais/psicológicos ^31^ .

A variável de acesso aos serviços de saúde não apresentou significância estatística no nosso modelo final, todavia destacamos as representações masculinas de saúde-adoecimento e cuidado e a busca por assistência à saúde. A elevada prevalência dos TMC no sexo masculino pode estar relacionada às barreiras para a presença masculina nos serviços de saúde, que conecta à estrutura de identidade de gênero (a noção de invulnerabilidade, a busca de risco como um valor), a qual dificultaria a verbalização de suas necessidades de saúde no contexto da assistência ^32^ .

Em cidades rurais, sobretudo da região Nordeste do Brasil, há poucos serviços direcionados aos cuidados em saúde mental e menor volume de recursos destinados ao diagnóstico e tratamento dos transtornos mentais. Para os casos considerados mais leves, o tratamento, na maioria das vezes, resume-se à assistência medicamentosa ou encaminhamentos a especialistas, mostrando deficiências na rede local de cuidados em saúde mental e as dificuldades dos profissionais dos serviços primários em lidar com essas formas de sofrimento ^25^ .

Embora a Estratégia de Saúde da Família tenha trazido inovações em termos de estrutura e processo de trabalho quando comparada à atenção primária à saúde convencional, ela ainda não foi capaz de proporcionar transformações na atenção em saúde mental, tais como a maior e melhor disponibilidade de profissionais e de recursos, a estruturação de fluxos próprios para a saúde mental, a escuta psicossocial, o acolhimento da alteridade e o trabalho integrado em rede ^33^ .

No presente estudo, a presença dos TMC esteve associada à utilização prévia dos serviços para tratamento de saúde mental. Estudos mostram que indivíduos que residem em áreas rurais e que possuem transtornos mentais, geralmente afirmam não procurar os serviços de saúde, devido às dificuldades de acessibilidade geográfica e pelas experiências não satisfatórias de acolhimento ^12 , 34^ . Todavia, a elevada prevalência de transtornos mentais como o uso abusivo do álcool ^35^ e o comportamento suicida ^36^ em populações que residem em áreas rurais, pode explicar a maior procura por esses serviços.

É importante considerar que a rede de saúde do município de Caicó é constituída por serviços de diferentes níveis de complexidade de atenção à saúde. No âmbito da saúde mental, o município dispõe de Centro de Atenção Psicossocial III e Centro de Atenção Psicossocial Álcool e Drogas e Residências Terapêuticas ^37^ . A maior prevalência de realização de tratamentos prévios relacionados à saúde mental entre os que possuem TMC pode ser um indicador de oferta da atenção à saúde mental.

Quanto à autoavaliação do estado de saúde, estudos ^30 , 38^ mostram que indivíduos que possuem TMC tem baixa avaliação de sua própria saúde, corroborando os resultados do nosso trabalho. Em geral, esses indivíduos possuem outras comorbidades associadas aos TMC, o que pode gerar perda de qualidade de vida e influenciar na autoavaliação do seu estado de saúde ^39^ .

A prevalência dos TMC foi menor entre indivíduos a partir dos 60 anos. Esse achado é contrastante com os de outros autores ^6^ , de que há maior prevalência de TMC entre os idosos, com baixo nível de escolaridade e tabagistas. Estudos internacionais em população geral mostram prevalências dos TMC em idosos variando entre 32,4%, na Etiópia ^40^ e até 51,8%, na Dinamarca ^41^ . No Brasil, o estudo realizado em Ibicuí-BA indicou a prevalência de 55,8% dos transtornos mentais na população idosa ^6^ ; em Campinas-SP, foi de 29,7% ^42^ . Há um gradiente crescente de TMC com o aumento da idade ^38^ , pois no processo de envelhecimento, muitos fatores podem contribuir para tal aumento, entre eles a elevada presença de morbidades e incapacidades, condições precárias de vida, eventos estressantes de vida, isolamento social e dificuldades econômicas.

Em relação à raça ou cor da pele, apesar de não ter sido um fator associado ao desfecho na análise multivariada, é importante destacar que, em termos culturais e sociais, a raça negra ainda está ligada a vulnerabilidades e às piores condições econômicas, de escolaridade, de trabalho e moradia ^43^ . Inclusive, estudos já demonstram disparidades raciais no acesso ao tratamento de transtornos mentais, no qual a população negra possui menor acesso aos serviços de saúde mental ^44 , 45^ . É importante lembrar ainda o quanto as questões étnico-raciais são escassamente abordadas nos estudos sobre a saúde e pouco se discute o impacto que as discriminações e opressões exercem sobre a saúde mental ^46^ .

Neste estudo, ter tido perda de produção foi um fator associado aos TMC. Um estudo ^47^ identificou que, em escala global, as perdas na produção pelo clima ou pragas reduzem as safras em até 40%, destacando-se as perdas de arroz (de 25% a 41%), milho (de 20% a 41%), batata (de 8% a 21%) e soja (de 11% a 32%).

O clima é o principal fator responsável pelas oscilações e frustrações das safras agrícolas no Brasil. Essa dependência das condições pluviais é particularmente sentida na Região Nordeste, em sua porção semiárida, área na qual está localizado o município de Caicó. Esse pedaço do território nordestino é formado por um conjunto de espaços que se caracterizam pelo balanço hídrico negativo, resultantes das precipitações médias anuais inferiores a 800 mm, insolação média de 2.800h/ano, temperaturas médias anuais que variam de 23ºC a 27ºC, evaporação de 2.000mm/ano e umidade relativa do ar média em torno de 50% ^48^ .

Na região do Seridó Potiguar, as condições climáticas naturalmente rigorosas, aliadas às pressões antrópicas – pecuária extensiva, agricultura de sequeiro (praticada nos meses de chuva na região) com culturas e técnicas inadequadas, indústria extrativista da cerâmica e mineração resultam em áreas extremamente degradadas e reduzem a capacidade adaptativa dos agricultores às mudanças climáticas futuras, aumentando sua vulnerabilidade. Todos esses fatores afetam negativamente a produção agrícola familiar já fragilizada nessa região ^49^ .

Nos municípios nordestinos, os agricultores inscritos no Programa Nacional de Fortalecimento da Agricultura Familiar (PRONAF) e que se enquadravam na linha de crédito do PRONAF-B (renda de até 20 mil reais anuais), possuem acesso a um programa de crédito financiado pelo Banco do Nordeste do Brasil desde o ano de 2005, o Programa de Microcrédito Rural Orientado e Acompanhado, denominado Agroamigo ^50^ .

Quando há perda de produção, sem o retorno financeiro esperado por aquela safra, os produtores veem suas dificuldades financeiras aumentarem, sem condições de quitar as dívidas, o empréstimo bancário ou mesmo para o sustento da família, fatores que irão impactar diretamente na saúde mental dos trabalhadores rurais. As dívidas financeiras estão relacionadas a vários problemas de saúde, particularmente à saúde mental ^51^ . Em Caicó-RN, embora a maioria das famílias rurais sobrevivam da agricultura, a disponibilidade de financiamento existe apenas para uma parte dessas famílias, uma vez que boa parte desses não possuem implementos agrícolas para manejo e comercializam a produção em agroindústrias, diretamente ao consumidor e aos varejistas ^29^ .

É importante destacar também que a estrutura fundiária do Seridó potiguar é baseada no grande número de área destinado às grandes propriedades, distribuídos para um baixo número de imóveis. O Seridó Oriental e Ocidental do Rio Grande do Norte é o que possui o maior número de grandes propriedades e municípios com estrutura fundiária concentrada, com destaque para o município de Caicó ^52^ . Na região do Seridó, existem 9.970 estabelecimentos voltados para agricultura familiar, o que representa 80,6% das propriedades agrícolas da região. Esses imóveis ocupam, porém, apenas 24,2% da área agrícola total ^14^ .

Associado à concentração de terra, o intenso processo de desertificação, pelo qual o semiárido nordestino passa, leva à degradação do solo, à redução da capacidade produtiva da agropecuária, à redução da renda agropecuária e à deterioração das condições sociais da população local ^52^ . Fatores como o tamanho reduzido das propriedades, a falta de recursos e de assistência técnica e o baixo nível de escolaridade limitam consideravelmente a capacidade de resiliência da agricultura familiar. Assim, em períodos de ocorrência de secas, estiagens ou enchentes, a produção agrícola é altamente prejudicada. As alternativas de adaptação encontradas por agricultores passam pela: redução da produção agrícola, migração dos jovens para as áreas urbanas do estado e obtenção de renda fora da propriedade ^14^ .

Nas últimas décadas, é inegável a melhora dos indicadores sociais do país, especialmente nas zonas rurais, como a queda nos índices de desigualdade, de pobreza e extrema pobreza, crescimento da renda *per capita* , bem como naqueles relativos à moradia, educação, saúde e lazer. Entretanto, a situação ainda é alarmante quando se considera, por exemplo, que um em cada quatro brasileiros que vivem no campo está em situação de pobreza extrema ^12^ .

Quanto à ingestão de bebida alcoólica, fazer uso abusivo do álcool foi associado à maior prevalência de TMC. O consumo moderado de álcool pode ter um impacto psicológico positivo, com alívio do sofrimento emocional e ser eficaz para lidar com situações sociais e de ansiedade ^53^ . Todavia, os transtornos mentais e o uso abusivo de álcool têm maior impacto negativo em estratos populacionais mais desfavorecidos socioeconomicamente, devido às situações de vulnerabilidade em que se encontram e aos poucos recursos sanitários com que contam. Em realidades que envolvem áreas de assentamentos rurais no Brasil, pelas duras condições de vida, de trabalho, pobreza, isolamento social e baixa cobertura de serviços públicos que dispõem, a população rural fica mais vulnerável à emergência de quadros suscetíveis ao desenvolvimento dos TMC e agravos quanto ao consumo abusivo de bebidas alcoólicas ^54^ .

O consumo abusivo de álcool está associado ao crescimento da taxa de mortalidade e morbidade, devido ao seu potencial de causar doenças e está relacionado à perda de qualidade de vida ^55^ . Em estudo realizado ^6^ a prevalência dos TMC em idosos foi mais elevada entre os que consumiam bebidas alcoólicas regularmente (61,5%), que relataram fumar atualmente (58,3%) e entre os que relataram que já tinham fumado em algum período da vida (56,1%).

Apesar deste estudo não ter encontrado relação entre os transtornos mentais e o hábito do tabagismo, outros estudos apontam para a existência de associação entre eles, com base na ação do tabaco sobre o sistema neurotransmissor, influenciando o quadro psicopatológico e causando reações, tais como: sintomas emocionais, problemas de conduta e problemas de relacionamento ^54^ . Em estudo longitudinal realizado na Índia com 2.094 mulheres com idade entre 18 e 45 anos, constatou-se que tabagistas apresentaram uma incidência de transtornos mentais quatro vezes maior do que as não fumantes ^55^ . Além disso, a nicotina atua na desregulação no eixo hipotálamo-pituitária-adrenal e no aumento da ação do cortisol no cérebro, uma alteração que é característica dos sintomas depressivos, portanto, o tabagismo poderia gerar maior suscetibilidade para o desenvolvimento dos TMC ^27^ .

Os resultados obtidos neste estudo devem ser avaliados com cautela, devido às possíveis limitações do estudo transversal. É possível que tenha ocorrido viés de seleção, uma vez que os trabalhadores selecionados para a pesquisa deveriam estar filiados ao sindicato local no momento da pesquisa. Outra limitação está no instrumento utilizado na coleta de dados, o SRQ-20, que não foi autoaplicado. A maioria dos participantes da pesquisa tiveram dificuldades, provavelmente devido à baixa escolaridade, para compreender as questões feitas, obrigando uma maior participação das pesquisadoras. Essas intervenções podem ter gerado vieses de informação.

Este estudo sugere que os TMC têm associação com as variáveis sexo, faixa etária, autoavaliação do estado de saúde, uso abusivo de álcool, submissão anterior a tratamento para saúde mental e perda de produção agrícola. Esses achados podem fortalecer o campo de discussão sobre a determinação social da saúde mental, debate ainda incipiente entre as equipes de saúde e formuladores de programas e políticas de saúde. Tais resultados corroboram a literatura que demonstra que o sofrimento mental entre agricultores e trabalhadores rurais é um problema de grande magnitude. Ações que visem à melhoria das condições de vida e trabalho podem ser benéficas para a manutenção e fortalecimento das condições de saúde mental dessa população.

No contexto da pandemia de covid-19, é importante considerar as condições de saúde mental da população diante dos múltiplos reflexos que essa pandemia tem causado, provocando mudanças significativas no quadro de saúde mental da população em âmbito mundial ^56^ . Diante do problema identificado no presente estudo, é preciso uma atenção especial às demandas psicológicas desses trabalhadores rurais que possam se agravar em decorrência da pandemia, o que torna urgente a proteção à saúde mental desse grupo vulnerabilizado.

Nossos achados indicam a importância de fortalecer a rede de atenção à saúde, principalmente nos serviços de atenção psicossocial, visando o desenvolvimento de estratégias preventivas para esses grupos e a diminuição dos índices de adoecimento mental. Conclui-se que o acompanhamento periódico da saúde mental desses agricultores é de suma importância para identificação precoce dos problemas que inferem na saúde do trabalhador, logo, cabe aos gestores propor estratégias não apenas de monitoramento, mas de intervenção precoce de forma a minimizar os sintomas e manifestações presentes, prevenindo a ocorrência, deteriorações emocionais e agravos psicológicos.
